# Current Tick Control Strategies and Prospects for Using Nanotechnology as an Efficient Alternative—A Review

**DOI:** 10.3390/vetsci12020163

**Published:** 2025-02-12

**Authors:** Rafaela Regina Fantatto, João Vitor Carvalho Constantini, Flávio Augusto Sanches Politi, Rodrigo Sorrechia, Camila Cristina Baccetti Medeiros, Marcela Tavares Luiz, Gervásio Henrique Bechara, Ana Carolina de Souza Chagas, Marlus Chorilli, Rosemeire Cristina Linhari Rodrigues Pietro

**Affiliations:** 1 Departament of Drugs and Medicines, School of Pharmaceutical Sciences, São Paulo State University UNESP, Rodovia Araraquara-Jaú Km 1, Araraquara 14800-903, SP, Brazil; rrfbio@hotmail.com (R.R.F.); joao.constantini@unesp.br (J.V.C.C.); flaviopoliti@hotmail.com (F.A.S.P.); rodrigo.sorrechia@unesp.br (R.S.); camila.medeiros@unesp.br (C.C.B.M.); marlus.chorilli@unesp.br (M.C.); 2 Graduate Program in Animal Science—PPGCA, Pontifical Catholic University of Paraná, Rua Imaculada Conceição, Curitiba 80215-901, PR, Brazil; gervasio.bechara@pucpr.br; 3 Southeast Livestock Unit, EMBRAPA—Brazilian Agricultural Research Corporation, Rodovia Washington Luiz, Km 234, São Carlos 13560-970, SP, Brazil; carolina.chagas@embrapa.br

**Keywords:** tick control, nanotechnology, acaricides, plant extracts, resistance management, environmental impact

## Abstract

Ticks pose significant challenges to public and veterinary health, acting as vectors of several diseases that affect animals and humans. Traditional chemical control methods, such as pyrethroids and organophosphates, have led to increasing resistance and environmental contamination, highlighting the need and urgency for alternative strategies. This review explores contemporary approaches to tick control, emphasizing plant-derived acaricides and their integration with nanotechnology. Plant extracts, known for their acaricidal properties, disrupt several biological processes in ticks, reducing reproduction and survival rates. The advent of nanotechnology offers promising advances in increasing the efficacy of these natural extracts. Nanoparticles add properties to the systems where they act by improving the stability, bioavailability, and targeted delivery of plant-derived compounds, potentially overcoming the limitations of traditional acaricides. This synthesis of current knowledge highlights the potential of combining plant extracts with nanotechnology to develop sustainable and effective tick control solutions, addressing issues of acaricide resistance as well as environmental concerns. The review also identifies research gaps and suggests directions for future studies to optimize the application of nanotechnology in tick management.

## Simple Summary

Ticks represent a major public and veterinary health problem, as they are vectors of several diseases that affect humans and animals. Traditional control of these ectoparasites is based on the use of synthetic chemical acaricides, but the increasing resistance to these molecules has limited control options, in addition to generating environmental impacts. Therefore, new approaches and strategies must be explored to ensure more effective and sustainable control. This article presents a comprehensive review of contemporary tick control methods, with an emphasis on the use of plant-derived acaricides and their integration with nanotechnology. Plant extracts have acaricidal properties that interfere with the biological processes of ticks, and nanotechnology emerges as a promising tool to increase the stability, bioavailability, and targeted release of these natural compounds, enhancing their efficacy. The combination of plant extracts with nanotechnology presents a viable and efficient alternative to overcome the limitations of conventional acaricides.

## 1. Introduction

Ticks are a major veterinary and public health problem, being one of the most important vectors of microorganisms and potentially negatively impacting animal production and health due to the direct effect of massive blood depletion or by transmitting infectious agents through their salivary glands [1], such as viruses, bacteria, rickettsiae, and protozoa [2]. They are spread around the world and, depending on the species, can be found in highly varied habitats, from desert areas to extremely humid regions [3]. These ticks are classified into three different families, including Ixodidae, Argasidae, and Nuttalliellidae. The first family includes the most economically important species, known as hard ticks, which are so named due to the presence of a very rigid chitinous shield covering the entire dorsal surface of adult males [4].

The economic impacts caused by these arthropods encompass both animal and human health [3]. In the veterinary field, the economic impacts on animal production are noteworthy, due to direct damage (skin lesions, blood depletion, and decreased productivity) and indirect damage (infectious conditions where ticks are vectors) caused to the animals [5]. In human health, ticks stand out as vectors of different tick-borne diseases, such as Lyme disease, Babesiosis, Anaplasmosis, Tularemia, and Rocky Mountain Spotted Fever [6]. Lyme disease and tick-borne encephalitis stand out as two of the main diseases transmitted by ticks. The species *Ixodes ricinus* acts as the main vector of these diseases, and its distribution is influenced by climate changes, which affect both the biology of the vectors and the transmission of the diseases [7,8,9]. Lyme disease is caused by the bacterium *Borrelia burgdorferi*, while tick-borne encephalitis is caused by the virus Tick-Borne Encephalitis Virus [9].

To deal with this parasite and minimize the damage caused, farmers are adopting measures that include the use of conventional chemical compounds such as pyrethroids, formamidines, macrocyclic lactones, thiazolidines, organophosphates, and phenylureas [10]. However, this approach often proves to be inefficient and especially unsustainable, leading to issues related to tick resistance to the synthetic agents used [11]. In addition to the challenges posed by the resistance of mites to acaricides, other issues arise, such as the environmental residues generated by synthetic products, their persistence as a source of environmental contamination, and the risks that these molecules pose to non-target organisms. These additional factors constitute significant challenges in the use of synthetic acaricides [12].

Safe and efficient tick control has become essential not only for agricultural productivity and human health but also for companion animals in the veterinary sector [13].

Due to this, there is a growing adoption of alternative measures to chemotherapeutic drugs in the control of ectoparasites, aiming to reduce parasite resistance and seek alternatives less harmful to animals and the environment [14], and in an attempt to find safe and efficient compounds with tick repellent and/or acaricidal properties, research on plant extracts traditionally used in tick control has grown in recent years, as seen in many reviews [15,16,17,18,19]. It is known that plants produce secondary metabolites as a means of defending themselves against pathogens, predators, and pests. The impact of these substances on ticks can result in the prevention of egg laying and hatching, inhibition of growth hormones, interference in reproduction and sexual communication, and blocking of chitin formation [13].

Plants are a source of several metabolites used in the health and environmental sectors, in addition to being widely used in commercial and pharmaceutical products. This wide variety of compounds produced can be divided into five main categories according to their action: growth regulators, nitrogenous compounds, phenolics, proteinase inhibitors, and terpenoids also present in essential oils. Essential oils are concentrated volatile aromatic liquids derived from plants and can be obtained from flowers, leaves, seeds, bark, twigs, bark, wood, roots, underground stems, etc. [20]. According to their chemical structure, these essential oils can be divided into phenols, terpenoids, aldehydes, ketones, ethers, epoxides, and other compounds [21]. Essential oils are frequently used in many products that have varied applications, such as sterilization, fungicides, antiparasitics, and insecticides, as well as pharmaceutical and cosmetic uses [22]. These compounds, as terpenoids, function as repellents, feeding deterrents, or toxic agents for phytophagous insects [13,23]. Studies have also demonstrated the efficiency of using thymol and carvacrol, components derived from essential oils, in combination, in a 1:4 ratio of thymol-carvacrol increased insecticidal activity reported against *Culex pipiens* larvae [24]. This combination also demonstrated high acaricidal activity against *Ixodes ricinus*, with both compounds achieving more than 90% repellency [25]. Furthermore, thymol and carvacrol together were effective against *Amblyomma sculptum* and *Dermacentor nitens* larvae, showing moderate synergistic effects [26].

The use of plant-derived acaricides is of utmost importance as an effective means of protecting livestock, especially in regions where agriculture and livestock are a significant part of the local economy. Extracts from various plant species have been reported to have harmful effects on ticks [27].

While natural product extracts are especially important for mite control, the use of innovative technologies should enable scientific advances in this area. Nanomaterials are a category of substances with dimensions ranging from 1 to 100 nm. However, the Food and Drug Administration (FDA) has not established a regulatory definition for “nanomaterial.” For drug products that contain nanomaterials, the FDA may consider products having one or more dimensions up to 1000 nm (or 1 micron) [28]. This category of particles has gained recognition in technological advancement due to their multifaceted properties compared to other particles, resulting in remarkable applications in all areas of technology [29]. The use of nanostructures associated with plant extracts is based on the goal of increasing the stability of components, reducing the occurrence of resistance of natural products, and decreasing cytotoxicity against other organisms, which consequently improves their bioavailability and acaricidal action [30].

This review aimed to explore and synthesize contemporary tick control strategies using plant extracts associated with nanotechnology. By examining the effectiveness of various approaches, our goal was to identify gaps in current knowledge. Additionally, we addressed the potential of nanotechnology as a promising and efficient alternative for tick control, analyzing the latest developments and their practical implications. The databases PubMed, Web of Science, Science Direct, and Google Scholar were used.

## 2. The Ixodidae Tick

There are approximately 867 known species of ticks in the world. Of these, about 10% are recognized as vectors for a wide range of pathogens that affect animals and humans [31].

Only in Brazil until 2021, there were 76 different species of ticks, among which 25 belonged to the Argasidae family and 51 to the Ixodidae family [32]. Ticks of the Ixodidae family, known as hard ticks, have a dorsum partially or completely covered by sclerotized layers, a characteristic that allows for differentiation between female and male adults of some species. It is the most numerous family, with between 702 and 713 species around the world, and the most significant in terms of medical and veterinary importance [4]. This family is subdivided into two groups:his family is subdivided into two groups: Prostriata and Metastriata. The Metastriata group includes the following subfamilies and their respective genera: *Amblyomminae* (*Aponomma* and *Amblyomma*); *Haemaphysalinae* (*Haemaphysalis*); *Hyalomminae* (*Hyalomma*); and *Rhipicephalinae* (*Cosmiomma*, *Dermacentor*, *Rhipicentor*, *Anomalohimalaya*, *Nosomma*, *Rhipicephalus*, *Boophilus*, and *Margaropus*). The Prostriata group includes the subfamily Ixodinae, with the genus *Ixodes* [33]. Although they are commonly recognized vectors for the transmission of pathogenic agents responsible for Lyme disease, Babesiosis, Anaplasmosis, Tularemia, and Rocky Mountain spotted fever, ticks from the Ixodidae family have been identified in recent studies as potential reservoirs for the transmission of various other diseases, as described in Table 1.

Data from the Center for Disease Control and Prevention (CDC) on major tick-borne diseases in the United States, along with the described research, highlight a growing increase in the number of confirmed cases year after year. This trend is likely correlated not only with globalization and the increase in global warming [45], but primarily with the inefficacy of ectoparasite control using currently available chemical products due to the development of resistant strains [46].

## 3. Generation of Resistant Strains and Control Strategies

Generally, resistance is recognized as a failure of a chemical product in controlling a certain parasite; however, the formal definition of resistance is the occurrence of a change in the natural susceptibility of target species to a specific chemical agent or to a class of chemical agents [47]. The development of resistance is an evolutionary process that arises through genetic selection [48]. Various species of arthropods have naturally survived adverse conditions through a gradual adaptation process to maintain homeostasis in ecosystems. Before the administration of a new acaricide, alleles that confer resistance are rare, occurring in about 1 in every 1 million (or more) individuals [49]. When the acaricide is used, resistant individuals have a selective advantage, i.e., they survive the treatment, reproduce, and give rise to a new generation of ticks that are also resistant. As new applications of the product are made, the susceptible portion of the population is eliminated, and there is a predominance of resistant individuals, reducing the effectiveness of the product. The time for resistance to develop appears to be governed by the degree of dominance of the alleles involved and the frequency and quality of treatments [50].

The speed at which resistance develops in a population depends primarily on the initial frequency of genes conferring resistance, the intensity of selection, the degree of dominance of the genes, and the relative capacity of the genotype [51]. There is an exponential pattern of development between the discovery of new acaricides and the development of species resistant to these new products, characterized over the years by occurring in increasingly shorter periods. For the development of resistance to dichloro-diphenyl-trichloroethane (DDT), the first records of cases of strains insensitive to the chemical pesticide were reported about 6 years after its introduction to the market, while for lindane, organophosphates, carbamates, and synthetic pyrethroids, it took 5, 4, 2.5, and 2 years, respectively [52].

Obaid and colleagues (2022) highlight the importance of understanding the involvement of causes that directly influence the development of resistance evolution, which can occur in different ways, such as genetic, biological, or operational factors. Parasites resistant to drugs are more likely to be selected if their populations are exposed to sub-therapeutic concentrations through the application of inadequate treatment regimens, especially with unregulated products. Unlike genetic factors, operational factors can be controlled through proper management, educating operators about the rational use of acaricides [53]. In addition to the aforementioned criteria, four mechanisms of resistance have been identified and agreed upon [54]: 

Insensitivity of action sites: It is primarily characterized by a nucleotide mutation in the coding region of a gene. This mutation can result in an amino acid change and consequently a three-dimensional alteration in the protein(s) forming the receptor(s). This structural change can reduce or block the ability of the molecule to bind to the action site, resulting in resistance [53]. For example, organophosphate and carbamate insecticides inhibit acetylcholinesterase (AChE). Arthropod populations become resistant to these compounds when individuals within the population develop a structurally modified AChE enzyme that allows them to survive exposure to organophosphate and carbamate [55].

Metabolic resistance: This type of resistance is characterized by an increase in the capacity of resistant individuals to detoxify and/or eliminate the acaricide products used in treatment. This increase can result from: (a) increased expression of enzymes responsible for drug metabolism, mainly cytochrome P450 monooxygenases (P450), esterases, and glutathione S-transferases (GST); and (b) increased specificity of these enzymes for the insecticide, facilitating its detoxification [56].

Behavioral resistance: This occurs when the arthropod modifies its behavior to avoid contact with the pesticide, such as a greater tendency to move away from a treated surface or area. It is often difficult to assess whether behavioral evasion is genetic or adaptive [57].

Cuticular penetration resistance: This is a modification of the exoskeleton of the arthropod to inhibit or delay the penetration of the chemical product and generally involves the concentration of lipids that facilitate or retard the penetration of the pesticide through this structure. Reduction in cuticular penetration delays the absorption of an insecticide. This is usually not remarkably effective unless combined with other resistance mechanisms [57].

The diagnosis of acaricide resistance is necessary to identify the problem, develop strategies for controlling the situation, adopt effective tactics to prevent the emergence of resistance, prevent the spread of resistant ticks, and save funds. According to [58], there are certain fundamental requirements that need to be met in the development or selection of the ideal laboratory test. A basic requirement is that the test should be capable of detecting the resistance problem at an early stage of its development and, if possible, before it becomes a major control problem in the field.

Acaricide resistance is constantly monitored and reported, demonstrating an increase in the number of strains resistant to distinct types of active ingredients, making it difficult to control these ectoparasites, as described in Table 2.

## 4. Revolutionizing Tick Control Through Nanotechnology

Nanotechnology has received massive attention in recent years due to the outstanding physical and chemical properties in comparison with bulk materials. Examples of these unique features are physical strength, permeability, chemical reactivity, electrical conductance, optical effects, and magnetism [61]. These unique characteristics are related to their nanoscale size, high surface area, and composition, which make this technology promising for application in the biomedicine field, including the veterinary medicine field. One of the veterinary applications that has explored the potential of nanomaterials is acaricidal science, which has used nanotechnology to manage the development and growth of ticks, reducing the negative impact of these vectors on animal production and health [62,63].

The nanostructure systems investigated for acaricidal application can show different sizes, shapes, and compositions. These systems are commonly classified according to their composition as inorganic (e.g., gold, silver, mesoporous silica, and iron oxide nanoparticles), organic (e.g., polymeric nanoparticles, micelles, nanoemulsion, liposomes, and nanostructured lipid carriers), and hybrid nanoparticles (a mixture of inorganic and/or organic materials) [64]. The ticks’ management using these nanostructures can be made by the acaricidal property of some nanoparticles or by their ability to deliver pesticides, as discussed below [61,65,66].

### 4.1. Nanostructure Systems with Acaricidal Activity

Inorganic nanoparticles (e.g., silver, gold, zinc oxide, silica, titanium oxide nanoparticles) have been commonly investigated for their acaricidal activity, which makes them widely studied for this purpose (Table 3). The mechanism evolved with the acaricidal activity of some inorganic nanoparticles is not completely understood. A widely acceptable theory is the oxidative stress generated by them in arthropod tissues. Moreover, other mechanisms have also been reported, such as the alteration of lipids and proteins and the disturbance of ticks’ metabolism [67].

This control of arthropod development and growth by nanostructures depends intrinsically on their physicochemical characteristics, mainly particle size, shape, and composition. These properties hugely influence their ability to penetrate through the exoskeleton to promote their action in the management of these insects [64,77]. Therefore, the optimization of formulation parameters during the production steps is primordial for successful arthropod control. In the section below, the effect of the most studied nanostructures is exposed.

#### 4.1.1. Silver Nanoparticles

Silver nanoparticles (Ag NP) have been one of the inorganic nanoparticles most studied for acaricidal management. A study conducted by Nabil and colleagues (2023) [62] evaluated the acaricidal efficacy of Ag NP synthesized using the aqueous extract of *Commiphora molmol* (myrrh) and *Zingiber officinale* (ginger) against camel tick (*Hyalomma dromedarii*). Ag NPs were produced using laser ablation of a silver plate in each extract and showed spherical shapes and particle sizes of 14 nm and 15.3 nm for *C. molmol* and *Z. officinale* extracts, respectively. The efficacies of these formulations were evaluated through the adult immersion tests (AITs) and indicated that Ag NPs produced using *C. molmol* and *Z. officinale* extracts showed higher acaricidal activity than both extracts isolated, with a lethal dose (LC_50_) of 2.38 and 4.12% after fifteen days post-treatment, respectively. These results indicated the efficacy of these eco-friendly formulations for tick’ control [78].

#### 4.1.2. Zinc Oxide Nanoparticles

Abdel-Ghany and colleagues (2022) biosynthesized zinc oxide nanoparticles (ZnO NP) from the aqueous extract of *Melia azedarach* to control *H. dromedarii* ticks. The green synthesis of ZnO NP was characterized by UV-Vis spectroscopy, Fourier transform infrared spectroscopy, scanning electron microscopy, and energy-dispersive spectroscopy. These analyses confirmed the development of ZnO NP with a spherical shape and size ranging between 18 and 42 nm. The efficacy of ZnO NP against *H. dromedarii* tick in several development stages (egg, ninthnimphy, and larva) was confirmed by performing the immersion tests, in which the LC_50_ was 11.6, 8.03, and 3.9 mg/mL, respectively. A reduction in the number of eggs laid by engorged females was also reported. Furthermore, ZnO NP showed an insignificantly toxic effect using a Swiss mice model, according to the hemogram, biochemistry, and histopathological analysis [79].

#### 4.1.3. Titanium Dioxide Nanoparticles

Rajakumar and colleagues (2015) investigated the larvicidal efficacy of titanium dioxide nanoparticles TiO_2_ NP against blood-feeding parasites, including *Rhipicephalus (Boophilus) microplus, Hyalomma anatolicum anatolicum*, and *Haemaphysalis bispinosa*. The TiO_2_ NP was produced using an eco-friendly approach through biosynthesis with the leaf aqueous extract of *Mangifera indica* L. Nanoparticles showed spherical and oval shapes, with sizes around 30 nm. The biological assay indicated that TiO_2_ NP had larvicidal activity, with an LC_50_ of 28.56, 33.17, and 23.81 mg/L for *R. microplus*, *H. anatolicum anatolicum*, and *H. bispinosa*, respectively [75]. These results indicated that TiO_2_ NP can control ticks’ development, possibly by the mechanisms previously cited.

### 4.2. Nanostructures as Drug Delivery Systems

In addition to some nanoparticles being applicable in tick management due to their biotoxic activity against ticks, nanotechnology can also be used for delivering pesticides. Generally, organic nanoparticles have been widely used for delivering pesticides, which include lipid-based (e.g., liposomes and nanoemulsions) and polymer-based (e.g., polymeric nanoparticles and micelles) nanosystems. The use of these nanosystems for delivering active molecules can present several advantages that assist in multiple ways with the acaricidal control and prevent environmental damage [64,80,81,82]. The most significant benefits of nanosystems for pesticide delivery include [77,81,83,84]:

- The encapsulation of pesticides in nanosystems can significantly enhance the dispersion of hydrophilic molecules, reducing the need for the addition of organic solvents and other solubilizers that can generate environmental damage and animal toxicity.
- The production methods most used avoid the use of hazardous organic solvents.
- Encapsulation enhances the stability of active molecules, given that it can avoid early deterioration by environmental conditions.
- Nanosystems can control the release of active molecules, providing a precise release kinetics
- The reduced particle size and the composition of nanosystems can improve pesticide permeation through arthropod exoskeleton

Thus, these advantages together enable an increase in the bioavailability of pesticides, allowing the use of low doses to promote an efficient acaricidal activity. In addition, reducing the effective dose and the use of solvents can reduce the toxicity of pesticides to animals and their damage to the environment [66,68]. In the section below, the effects of lipid- and polymer-based nanoparticles are discussed.

#### 4.2.1. Polymer-Based Nanoparticles

Polymeric-based nanoparticles are one of the most used nanostructures for controlled release of active molecules. These nanosystems are composed of natural (e.g., chitosan, alginate, and zein) or synthetic polymers (e.g., poly-ε-caprolactone, poly(D,L-lactic acid), and poly(lactic-co-glycolic acid)) and prepared in different structures, including nanocapsules, nanospheres, micelles, dendrimers, nanogels, and nanofiber. Among them, nanospheres are the most popular structures used for tick’ management, mainly those composed of natural polymers, which are considered environment-friendly polymers [83,85].

Figueiredo and colleagues (2023) produced zein nanoparticles to control the impact of the cattle tick *Rhipicephalus microplus* [86]. Zein is a corn protein that has been used to produce polymeric nanoparticles due to its amphiphilic behavior, which enables the encapsulation of hydrophilic and hydrophobic molecules. Due to its encapsulation capacity, the authors produced three nanosystems encapsulating cypermethrin, chlorpyrifos, and a plant compound (citral, menthol, or limonene). The zein nanoparticles activity was evaluated in the larval stage and in engorged females, and its efficacy was compared with a commercial acaricide (Colosso^®^). All nanoformulations caused larvae mortality higher than 80% at concentrations above 0.029 mg/mL, while Colosso^®^ resulted in 71.9%. Furthermore, nanoformulations exhibited higher acaricidal efficacy on engorged females than the commercial formulation (39.4%), with maximum value when zein encapsulates limonene as a plant component (60.1%). In addition, all then demonstrated lower toxicity to non-target nematodes, indicating the potential of developed formulations against ticks [86].

Berni and colleagues (2022) developed another polymeric nanoparticle composed of chitosan-coated poly-ε-caprolactone for delivering acaricidal substances (amitraz and fluazuron) against *Rhipicephalus (Boophilus) microplus* [69]. In this case, chitosan was used to provide mucoadhesive properties to polymeric nanoparticles. The developed formulation showed a particle size near 275 nm, a positive surface charge, and encapsulation efficiency above 75%. The in vitro cytotoxicity assay indicated that the polymeric nanoparticles decreased the cytotoxicity of the free amitraz and fluazuron, while the in vivo study indicated the ability of the formulation to protect bovines against *R. microplus*, which demonstrated the promising potential of the developed nanosystem [87].

#### 4.2.2. Lipid-Based Nanoparticles

Lipid-based nanoparticles are composed of biocompatible and biodegradable lipids. Generally, these nanostructures have great stability, generate high encapsulation efficiency of active molecules, and promote controlled release. In addition, the most common methods used to produce lipid-based nanoparticles are eco-friendly, avoiding the use of hazardous solvents. As polymer-based nanoparticles, those composed of lipids can also present different three-dimensional structures, which include solid lipid nanoparticles (SLN), nanostructured lipid carriers (NLC), liposomes, nanoemulsions, and microemulsions [83,88].

Figueiredo and colleagues (2022) developed two different types of lipid-based nanoparticles (SLN and NLC) associated with synthetic (cypermethrin and chlorpyrifos) and plant-derived (citral, menthol, or limonene) compounds for *R. microplus* control [89]. Nanoformulations showed a particle size from 286 to 304 nm, a monodisperse size distribution profile, a negative surface charge, and an encapsulation efficiency of about 98% for all active compounds. In addition, there was no significant difference in these physicochemical characteristics during the 120-day storage period, demonstrating the colloidal stability of all nanoformulations, which is important for their commercialization. The acaricidal action against *R. microplus* larvae was evaluated using NLC and SLN with and without plant-derived compounds. The results indicated that the addition of citral, limonene, or menthol improved acaricidal activity. Therefore, the authors suggested that combining natural and synthetic pesticides in nanosystems can overcome acaricidal resistance and help to control tick populations [88].

Ibrahium and colleagues (2022) produced a nanoemulsion as an eco-friendly nanoformulation for delivering d-limonene against *Rhipicephalus annalatus* and *Rhipicephalus sanguineus* ticks [89]. The nanoemulsion showed significant activity against both adult and larval stages of *R. annalatus* and *R.* sanguineus ticks and the ovicidal effect against *R. sanguineus* [90]. Another study has also demonstrated the acaricidal effect of a nanoemulsion containing a natural product (garlic oil), which induced complete mortality within 48 h of treatment [90]. Thus, both researchers demonstrated the potential of these eco-friendly lipid-based nanoparticles for tick control.

### 4.3. Challenges Faced by Nanotechnology Application

As mentioned before, nanotechnology has become an interesting strategy to control tick development and growth through the acaricidal activity of some nanomaterials or by delivering active molecules [61,64]. However, despite their numerous benefits against ticks, there is no nanotechnology product available on the market to the present date. The lack of these products on the market is related to several factors, including economic and environmental factors [91].

The development of nanosystems for acaricidal activity involves a long period, including the nanomaterial optimization process, scalability, and in vitro and in vivo assays to evaluate their efficacy and safety. All these steps require a high level of knowledge and financial investments, which can increase the cost of the product and make its commercial application a challenge. Moreover, the lack of regulation is another hindrance to the growth of nanotechnology for ticks’ management, and it needs to be improved to allow safe and effective products to reach the market [65,81].

Another important factor to be considered during nanosystems development is their environmental impact; it is extremely important to know the ecological hazard that the nanomaterials represent before making them available for the population. As mentioned, nanosystems can present unique physicochemical characteristics (e.g., particle size, surface charge, shape, and composition) that are responsible for their biological activity. However, these characteristics can also negatively impact the environment and non-targeted insects and animals. Thus, during nanosystems development, possible impacts on soil, surface water, groundwater, and non-targeted organisms (microorganisms, plants, animals, and insects) must be investigated [65].

## 5. Conclusions

Ticks represent a significant veterinary and public health concern, impacting the livestock production chain and transmitting pathogens to humans and animals. Conventional strategies, based on chemical acaricides, face limitations such as tick resistance, health risks to animal handling workers, and negative environmental impacts. Alternatives such as plant extracts and nanotechnology show enormous potential. Plant extracts contain active compounds that are effective against ticks, offering advantages over chemical compounds as they offer greater handling safety and hinder the emergence of resistance. Nanotechnology increases the stability and bioavailability of these compounds, reducing their toxicity. New studies may offer new routes and strategies in this important area. This review identified that the combination of these approaches offers a promising current solution for future development aimed at this field of innovation for tick control.

## Figures and Tables

**Table 1:** Human infectious diseases transmitted by ticks of the Ixodidae family.

Ticks	Hosts	Clinical Importance	References
*Ixodes ricinus, Ixodes persulcatus* *Ixodes scapularis*, *Ixodes cookei*, *Haemaphysalis longicornis*	Deer, Cattle, Sheep, Horses, Rodents, Dogs and Humans	Tick-borne encephalitis (TBE)/TBE virus (TBEV) subtypes Siberian, Far East and European.	[34]
	Deer, Cattle, Rodents, Dogs and Humans	Powassan encephalitis (POW)/POW virus (POWV).	[35]
	Humans and dogs	Boutonneuse fever (BF).	[36]
*Amblyomma americanum* *Ixodes scapularis*	Deer, cattle, rodents and humans	*Borrelia*, *Ehrlichia*, *Anaplasma*, *Babesia* and *Bartonella.*	[37]
	Deer, humans	Babesiosis, anaplasmosis and Lyme Disease.	[38]
*Dermacentor reticulatus*, *Ixodes ricinus* *Dermacentor andersoni* *Dermacentor marginatus*, *D. reticulatus* *Hyalomma scupense*; *H. anatolicum*	Small rodents	Tick-borne encephalitis virus (TBEV; family Flaviviridae).	[39]
	Deer, cattle, horses, elk, and occasionally, humans	Vector of *Rickettsia rickettsii*, the agent of Rocky Mountain spotted fever (RMSF), and more recently, various other rickettsial agents, including *Rickettsia peacockii*, *Rickettsia bellii*, and *Rickettsia rhipicephali.*	[40]
	Small mammals	Tick-borne lymphadenopathy/*Dermacentor*-borne necrotic erythema and lymphadenopathy (TIBOLA/DEBONEL) syndrome.	[41]
	Cattle	Theileriosis caused by the apicomplexan hemoparasite *Theileria annulata.*	[42]
*Hyalomma dromedarii; H. impeltatum* *Dermacentor variabilis*, *Amblyomma americanum*	Ruminants, primarily goats, sheep, and cattle; dromedary camels	Q fever caused by *Coxiella burnetii.*	[43]
	Humans, small and large mammals	Vector of *Francisella tularensis*, the causative agent of the zoonotic disease tularemia	[44]

Source: Prepared by the authors, 2024.

**Table 2:** Acaricide resistance reported up to 2022 in the Arthropod Pesticide Resistance Database [59] according to cases and ineffective active ingredients in different tick families.

Mites Species	Family	Number of Resistance Reported Cases	Number of Ineffective Active Ingredients
*Tetranychus urticae* *R.* *microplus* *Panonychus* *ulmi*	* Tetranychidae *	552	96
	* Ixodidae *	562	50
	* Tetranychidae *	203	48
*Panonychus citri* *Rhizoglyphus robini*	* Tetranychidae *	94	29
	* Acaridae *	23	22
*Tetranychus cinnabarinus* *Boophilus* *decoloratus* *Typhlodromus pyri* *Tetranychus* *mcdanieli*	* Tetranychidae *	37	19
	* Ixodidae *	29	16
	* Phytoseiidae *	20	13
	* Tetranychidae *	19	13
* Amblyseius * *fallacis*	* Phytoseiidae *	26	12

Source: Adapted from Rouck and colleagues (2023) [60].

**Table 3:** Nanoformulations used against ticks, detailing their preparation method, tick species tested, and efficacy.

Nanoparticles (Size)	Preparation Method	Tick Species (Larvae or Adults)	Efficacy	Ref.
CLI (267 ± 2.25 nm)	Thin hydration method	*Rhipicephalus annulatus*; *R. sanguineus*	CLI (5%) induced mortality (100%) in *R. annulatus* adult ticks with LC_50_ of 2.60%, whereas the LC_50_ of pure carvacrol was 4.30%. Carvacrol and CLI were shown to have a significant larvicidal action on both tick species, with LC_50_ of 0.24 and 0.21% against *R. annulatus* and 0.27 and 0.23% against *R. sanguineus*, respectively.	[68]
CS_PCLnp_Amitraz (275 ± 30 nm) CS_PCLnp_Fluazuron (295 ± 35 nm)	Nanoprecipitation	* Rhipicephalus (Boophilus) microplus *	The acaricide effect was even stronger when CS_PCLnp_Amitraz (same dose as for commercial products) and CS_PCLnp_Fluazuron (half of the amount for commercial products) were administered together	[69]
Ag NPs (27.2–55.3 nm) ZnO NPs (72.6–95.4 nm)	Chemical reduction method (Ag NPs) and hydrothermal method (ZnO NPs)	*Rhipicephalus (B.) annulatus*	The adulticidal activity of deltamethrin- ZnO NPs at different concentrations (0.25–2.0 ppm) induced a significant lethal effect on adult ticks compared to deltamethrin-Ag NPs at the same concentrations. The larvicidal efficacy of deltamethrin-ZnO NPs resulted in a complete larval mortality within 24 h of exposure, while deltamethrin-Ag NPs exhibited 100% immobility of larvae 48 h post-exposure	[70]
Ag NPs (nanoparticle size not informed)	Green synthesis using neem leaf extract (N-Ag); deltamethrin/neem (DN-Ag); 2,3 dehydrosalannol (DHS-Ag); quercetin dihydrate (QDH-Ag)	*Rhipicephalus (B.) microplus*	Larvicidal effect (100%) at 200 ppm (N-Ag), 50 ppm (DN-Ag), 85 ppm (DHS-Ag) and 200 ppm (QDH-Ag); adult mortality of 93.33% at 50 ppm and LC_50_ = 3.87 ppm for larvae and LC_50_ = 21.95 ppm for adults (DN-Ag)	[71]
ZnO NPs (21.32 nm)	Green synthesis using *Momordica charantia* leaf extract	*Rhipicephalus (B.) microplus*	LC_50_ = 6.87 ppm for larvae of *R. (B.) microplus*	[72]
ZnO NPs (20.0–65.0 nm)	Green synthesis using leaf extracts of *Lobelia leschenaultiana*	*Rhipicephalus (B.) microplus*	LC_50_ = 1.7 ppm for adults of *R. (B.) microplus*	[73]
Chitosan NPs (589 nm)	Phase inversion using anchored ethanolic extract of *Pouteria gardneriana*	*Rhipicephalus (B.) microplus*	The applicability of chitosan as an anchoring material and in the controlled release study was effective. There was higher release of the extract between pH 5 and 6, promoting slow release of the active ingredient, increasing the exposure time between the tick and acaricide	[74]
TiO_2_ NPs (30 ± 5 nm)	Green synthesis using leaf aqueous extract of *Mangifera indica*	* Rhipicephalus (B.) microplus; Hyalomma anatolicum* *anatolicum; Haemaphysalis bispinosa *	LC_50_ = 28.56 ppm for *R. (B.) microplus* larvae; LC_50_ = 33.17 ppm for *H. anatolicum* larvae; LC_50_ = 23.81 ppm for *H. bispinosa* larvae	[75]
TiO_2_ NPs (70 nm)	Green synthesis using aqueous leaf extract of *Solanum trilobatum*	*Hyalomma anatolicum* *anatolicum*	LC_50_ = 4.11 ppm for larvae of *H. anatolicum*	[76]

Source: Prepared by the authors, 2024.

## Data Availability

Not applicable.
